# Beneficial Effects of Motor Imagery and Self-Talk on Service Performance in Skilled Tennis Players

**DOI:** 10.3389/fpsyg.2022.778468

**Published:** 2022-05-06

**Authors:** Nicolas Robin, Laurent Dominique, Emma Guillet-Descas, Olivier Hue

**Affiliations:** ^1^Laboratory ACTES (EA 3596), Sport Sciences Faculty, University of Antilles, Pointe-à-Pitre, France; ^2^Laboratory ACTES (EA 3596), Sport Sciences Faculty, University of La Réunion, Saint-Denis, France; ^3^Univ Lyon, Université Claude Bernard Lyon 1, LVIS, Lyon, France

**Keywords:** motor imagery, self-talk, service, tennis, performance, service speed, efficiency

## Abstract

This research aim to investigate the effects of motor imagery (MI), focused on the trajectory of the ball and the target area, and self-talk (motivational function) before the actual strike on the performance of the service in skilled tennis players. Thirty-three participants (6 females and 27 males, *M*_age_ = 15.9 years), competing in regional to national competitions, were randomly divided into three groups: Control, MI, and MI + self-talk. They performed a pre-test (25 first service), 20 acquisition sessions (physical trial, physical trial + MI and physical trial + MI + self-talk), and a post-test similar to the pre-test, in match situations. The percentage of the first service, their speed, and the efficiency scores, evaluated by experts, were use as dependent variables and indicators of performance. While there was no difference in service speed ( *p* > 0.05), this study showed an improvement in the first service percentage and efficiency (all ps < 0.01) in the participants of the MI and MI + self-talk groups. Additionally, analyses revealed greater efficiency when MI was combined with self-talk compared to other conditions. It, therefore, seems advantageous for skilled tennis players to use MI and motivational self-talk before performing the first service balls.

## Introduction

Coaches and athletes widely recognize the potential effects of using mental strategies to improve performance (Crespo et al., 2006), especially in racket sports (Cece et al., 2020). The latter authors, in their recent systematic review, revealed that Motor Imagery (MI) was the most used technique in tennis. MI can be defined as the brain’s ability to recreate motor experiences in the absence of actual execution (Vasilyev et al., 2017). Many researchers have shown that MI and physical practice promotes motor learning and performance in the forehand (Guillot et al., 2015; Dana and Gozalzadeh, 2017); backhand (Hegazy et al., 2015; Turan et al., 2019), volley (Cherappurath and Elayaraja, 2017; Türk et al., 2019), service return (Robin et al., 2007) and service (Desliens et al., 2011; Fekih et al., 2020). For example, Dominique et al. (2021) recently showed that skilled tennis players who used MI intervention focused on the trajectory of the ball and the target, before serving, had higher percentage success and greater first service efficiency than the participants of the control group who only performed physical practice. Other researches also showed the beneficial effects of combining different strategies such as MI and self-talk, in mental training programs, in order to improve overall tennis performance (Mamassis and Doganis, 2004; Dohme et al., 2020). According to Latinjak et al. (2019), self-talk refers to over or covert verbalizations that the individual (e.g., tennis player) addresses himself or herself. A distinction is made between spontaneous (i.e., organic) or uncontrolled self-talk and goal-directed (i.e., strategic) self-talk (Latinjak et al., 2014; Van Raalte et al., 2016). The spontaneous self-talk statements relate to the activity (e.g., tennis match) that come to mind spontaneously and effortlessly. It generally concerns past events (e.g., “that was a bad shot”) or future outcomes (e.g., “I will win”). The goal-directed self-talk is a deliberate mental technique or strategies frequently used by athletes to optimize performance utilizing its cognitive function (Boudreault et al., 2016) or regulate emotions by means of its motivational function (Fritsch et al., 2020). Cognitive or instructional self-talk aims to improve performance by means of an attentional focus directed toward technique (e.g., “bending the knees”) or necessary motor actions (e.g., “getting back on the court”), whereas motivational self-talk can be employed to proactively and reactively regulate motivation, self-confidence, and emotion (e.g., “enjoy your game”) or to sustain effort (e.g., “I will play well in the next set”). Several studies support the effectiveness of self-talk in sports (for a review, see Hardy, 2006), and a few researchers showed comparable effects of the two self-talk functions (e.g., Hatzigeorgiadis and Biddle, 2008; Chang et al., 2014). However, in a precision motor football task, Hardy et al. (2015) showed greater performance, in skilled athletes, who used motivational compared to cognitive self-talk functions. The authors suggested that attention directed toward the execution of a technical gesture (e.g., service in tennis) could adversely affect the performance of a mastered skill (Porter et al., 2010) as proposed by some attentional theories (e.g., Masters and Maxwell, 2008).

Previous studies showed, in tennis, the beneficial effects of using MI (e.g., de Sousa Fortes et al., 2019; Dominique et al., 2021) or self-talk (e.g., Zourbanos et al., 2015) on service performance and successful game outcome; and other studies supported the combination of these two mental techniques, among others, in mental skills training program (e.g., Dohme et al., 2020). That’s why, this original study aimed to evaluate, in skilled tennis players, the influence of a combination of MI (based on the trajectory and the target to be reached) and controlled motivational self-talk, performed before the actual strike, on the performance of the first service balls in match play situations, which could be especially beneficial as it is the only shot that is not preceded by another leaving the server enough time to perform it. We hypothesized that this strategy (i.e., MI plus self-talk), should achieve greater performance than MI alone, which in turn should achieve higher performance than the absence of mental practice (i.e., control condition).

## Materials and Methods

### Participants

Thirty-three skilled tennis players (6 females and 27 males, *M*_age_ = 15.9 ± 2.1 years) volunteered to participate in the study. The participants competed in regional to national competitions (French second series) and played tennis for over 8 years (*M* = 9.5 ± 1.8 years) at the Team Run Elite Tennis Club Dionysien. The parents of the players signed a consent form to participate in this study, received details of their required involvement, and about their right to withdraw. They were randomly drawn into 3 groups: Control, (*N* = 11, 2 females and 9 males), MI (*N* = 11, 2 females and 9 males) and MI + self-talk (*N* = 11, 2 females and 9 males). This study, approved by the local ethics committee, was carried out per the Helsinki Declaration (ACTES-3596-0422).

### Material and Procedure

This study consisted of 3 phases performed in a green set tennis court during good weather. Week 1, participants performed a pre-test: 25 first service, in competitive situations (see Guillot et al., 2013 for a similar procedure). The speed (recorded using a radar Cordless MPH radar Gun Type R1000), the percentage of first services in and the efficiency (evaluated by two tennis qualified tennis coaches external to the research) of the first service served as dependent variables and indicators of performance (see Dominique et al., 2021 and Robin et al., 2021 for similar procedures). The second phase (i.e., acquisition), which consisted of 20 tennis sessions lasting 1.5 h (2 sessions per week), was carried out from week 2 to week 11. During each session, participants performed a standardized 30-min warm-up (i.e., jogging, sprint, controlled pop up rally, and 12 warm-up services) followed by 25 services under match play conditions by switching service box after each point and with 20-s rest between points. Participants in the Control group only performed physical trial and did not receive any special instructions. Before each service, those in the MI group were asked to perform MI using an external visual modality (i.e., seeing each other in third person as if they were being filmed with a camera) of a successful service by visualizing the trajectory of the ball as well as the target area in the appropriate service box (for a similar procedure see Guillot et al., 2013). Participants of the MI + self-talk group had to perform MI combined with motivational self-talk (e.g., “I/you can do it,” “come on,” “I feel good,” and “I will play well on the next point”) before serving. At the end of each MI session, participants of the two latter group had to self-assess the perceived vividness of visual images using an MI quality index consisting of a Likert scale ranging from 1 (“Unclear and faint mental representation”) to 6 (“Perfectly clear and vivid mental representation”; for a similar procedure, see Dominique et al., 2021). The third phase (i.e., post-test), performed in week 12, was identical to the pre-test. All the participants were filmed (Canon HD, Legria HF G25) during the pre-and post-tests.

### Data Analysis

For each test performed during pre- and post-test, the average speed (in km/h), the percentage (successful), and the efficacy scores of the first service were computed. For these dependent variables, ANOVAs were performed: 3 independent groups (Control vs. MI vs. MI + self-talk) × 2 phases (pre-test vs. post-test) with repeated measures on the second factor. Normality was checked (Kolmogorov–Smirnov test), α was set at 0.05 for all the analyses, effect sizes (ηp2) were indicated, and post-hoc analyzes were performed using Newman–Keuls tests.

## Results

### Imagery Ability

None of the participants of the MI and MI + self-talk groups reported having difficulty in performing MI (*M*_score_ = 5.1; SD = 0.9) and none of the participants of the Control group declared using MI during the 3 phases. The participants of the MI + self-talk group reported using self-talk during match circumstances.

### Speed

The ANOVA did not reveal a main effect of the group, *F*(2, 30) = 0.97, *p* = 0.89, $\eta_{p}^{2}$ = 0.01, and of the phase, *F*(1, 30) = 0.43, *p* = 0.39, $\eta_{p}^{2}$ = 0.01; nor significant interaction between the group and the phase, *F*(2, 30) = 0.78, *p* = 0.53; $\eta_{p}^{2}$ = 0.02 (Table 1).

**Table 1:** Mean (standard deviation) first service ball speed (km/h) for the control, MI and MI + self-talk groups during pre- and post-test (all ps > 0.05).

Group	Pre-test	Post-test
	Mean (SD)	Mean (SD)
Control	143.3(4.5)	146.1(5.2)
Imagery	145.7(5.7)	150.2(3.9)
MI + self-talk	146.9(6.1)	148.5(4.8)

*MI, Motor imagery; SD, Standard deviation.*

### Percentage of Success

**Figure 1:**
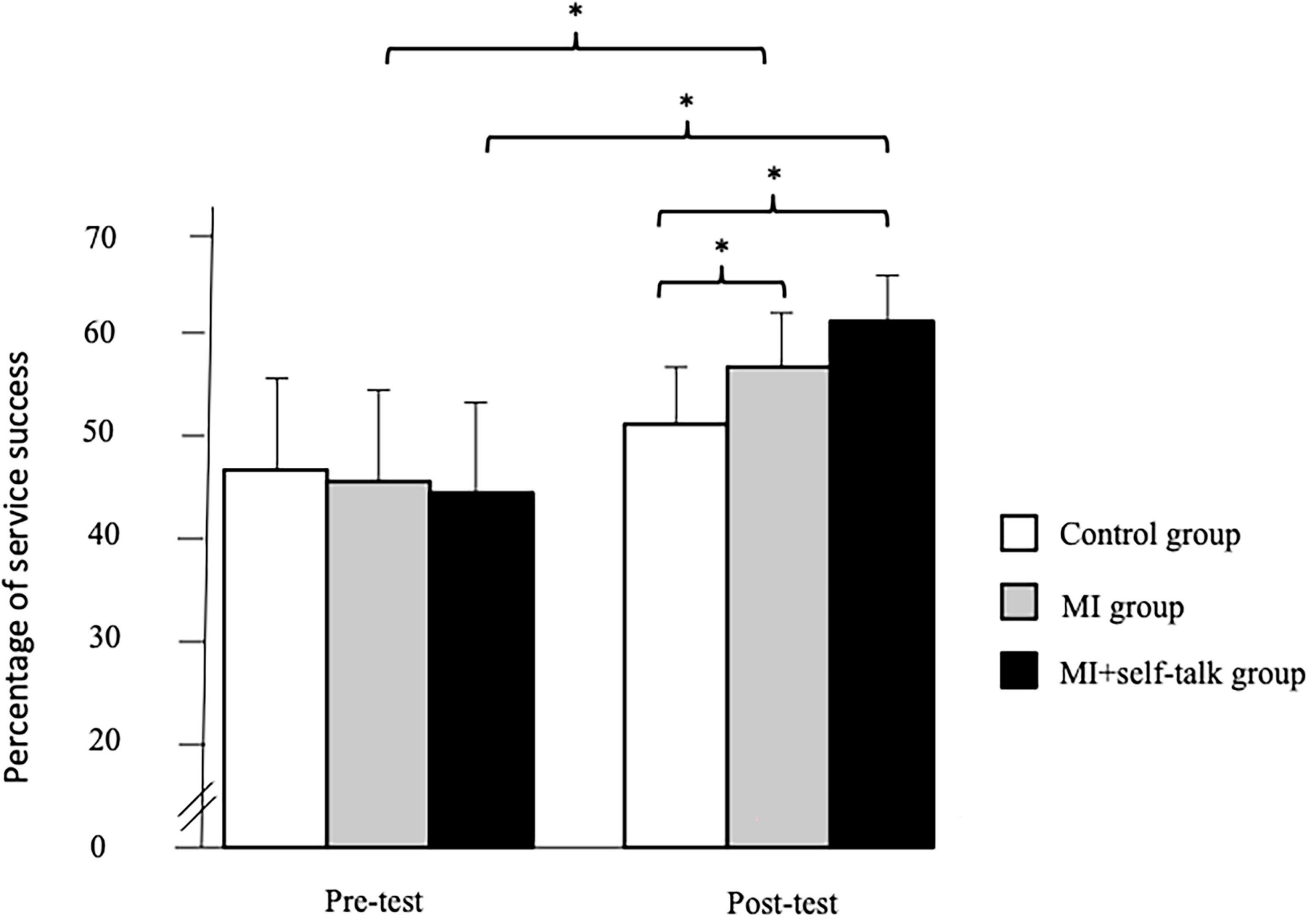
Significant interaction between the group and the phase (^*^p < 0.05) concerning the percentage of service success. Motor imagery (MI).

The ANOVA revealed a main effect of the phase, *F*(1, 30) = 59.28, *p* < 0.01, $\eta_{p}^{2}$ = 0.66, but an absence of main effect of the group, *F*(2, 30) = 0.57, *p* = 0.52, $\eta_{p}^{2}$ = 0.02. The analysis also revealed a significant interaction between the group and the phase, *F*(2, 30) = 5.48, *p* < 0.01, $\eta_{p}^{2}$ = 0.28. The post-hoc tests revealed that the participants of the MI and MI + self-talk groups increased their percentage of success of the first service from the pre- to the post-test and had greater performance than the Control group at the post-test (Figure 1).

### Efficiency

**Figure 2:**
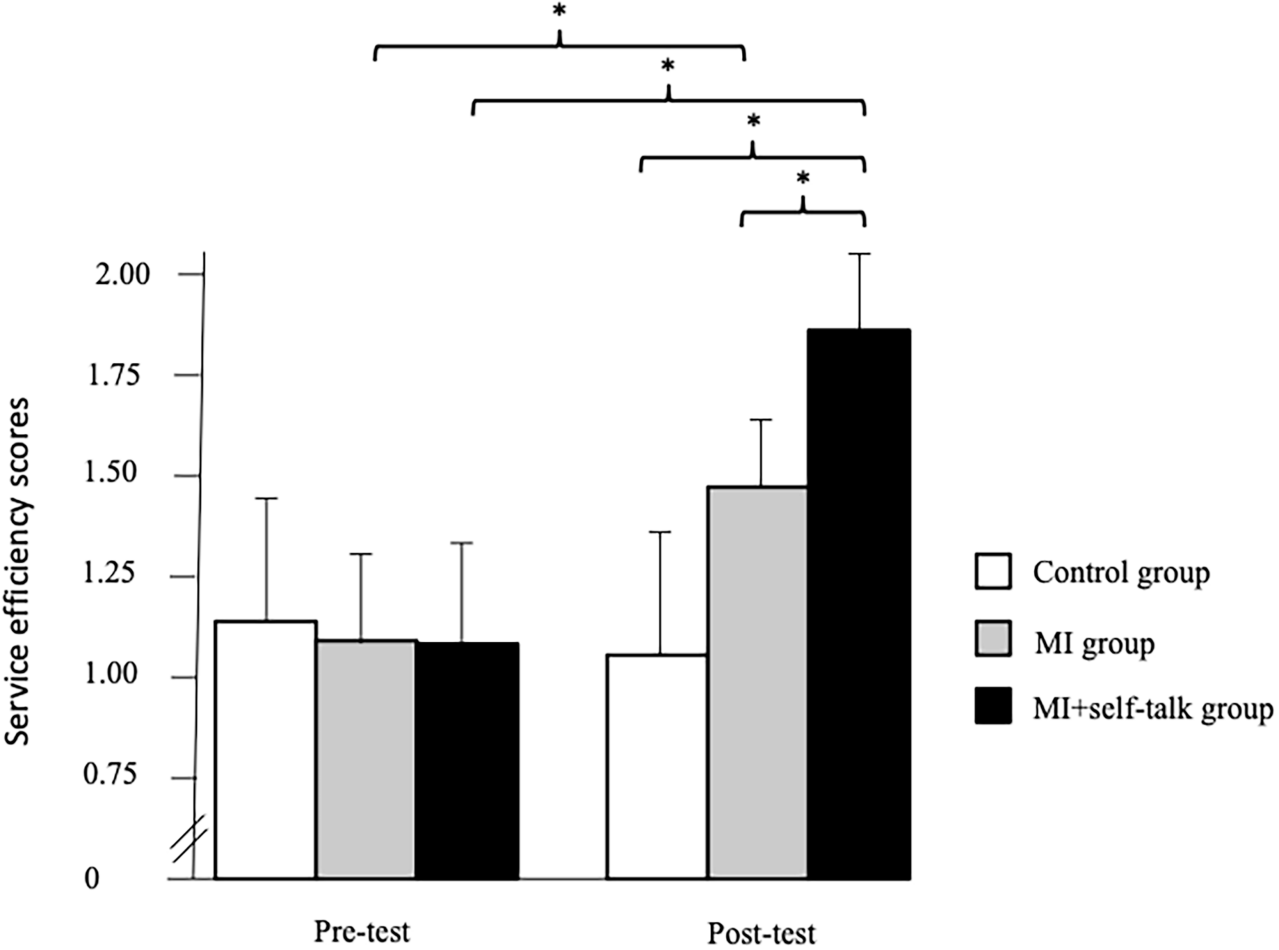
Significant interaction between the group and the phase (^*^p < 0.01) concerning the service efficiency scores. Motor imagery (MI).

The ANOVA revealed main effects of the phase, *F*(1, 30) =59.05, *p* < 0.01, $\eta_{p}^{2}$ = 0.66 and of the group, *F*(2, 30) = 5.45, *p* = 0.01, $\eta_{p}^{2}$ = 0.24. In addition, the analysis revealed an interaction between the group and the phase, *F*(2, 30) = 26.44, *p* < 0.01, $\eta_{p}^{2}$ = 0.47. As shown in Figure 2, the post-hoc tests revealed that the participants of the MI and MI + self-talk groups increased their first service efficiency scores from the pre- to the post-test and that the participants of the MI + self-talk had greater scores than the MI and Control group participants at the post-test.

## Discussion

This study aimed to assess the effects of a MI intervention focused on the trajectory of the ball and the target zone, combined or not with motivational self-talk, on the performance of the first service in skilled tennis players. The results of the study first revealed that using MI before serving improves the percentage of success and efficiency scores of the first service, while the performance of the control group remained stable confirming our hypothesis. These results confirm those of previous researchers that have shown beneficial effects of using MI interventions whatever the level of expertise of the practitioners (Toth et al., 2020). Indeed, while beneficial effects have been observed in children (e.g., Atienza et al., 1998), teens (e.g., Dana and Gozalzadeh, 2017) or adults (Cherappurath and Elayaraja, 2017) playing at a recreational level, others studies showed a positive effect of MI in teen with a regional level (Guillot et al., 2012), in young adults at collegiate level (Daw and Burton, 1994), in youth (Dohme et al., 2020) or young adult (Robin et al., 2007) elite players and even at a professional level (Mathers, 2017). More specifically, our results confirm the beneficial effect of MI on service performance in teen (Guillot et al., 2013; Türk et al., 2019) and young adults (e.g., Fekih et al., 2020; Dominique et al., 2021) skilled (i.e., national and elite) and international (e.g., Mathers, 2017) tennis players. However, the results of the current study did not show any improvement in service speed. Although participants in the MI group increased their service ball speed by just over 4 kilometers per hour, between pre-test and post-test, this difference was not statistically significant. As recently mentioned by Dominique et al. (2021), inconsistent results are reported in the literature. While some authors have observed an absence of change in the speed of service after MI intervention in skilled players (e.g., Guillot et al., 2012; Dominique et al., 2021), others showed an improvement among young tennis players (e.g., Mamassis, 2005; Guillot et al., 2013). This difference in results could on the one hand be explained by a possible weaker margin of progress for skilled players compared to beginners. On the other hand, it is possible that the duration of the acquisition phase, which consisted of 20 sessions over 3 months, should have been increased in order to be able to significantly improve the speed of the services of the participants in the current study.

Finally, the results of this study showed that the participants who performed MI combined with motivational self-talk (i.e., MI + self-talk group), had greater service performances (i.e., efficiency scores) than the participants of the other groups (i.e., Control and MI), supporting our hypothesis. The latter results confirm those of previous studies, which have shown the beneficial effects of combining different mental strategies (e.g., MI and self-talk) on tennis performance (Mamassis and Doganis, 2004; Dohme et al., 2020). In addition, these results seem to show the beneficial benefits of the motivational function of goal-directed self-talk (Hardy et al., 2015; Zourbanos et al., 2015; Boudreault et al., 2016; Fritsch et al., 2020), especially when this technique is combined with MI (Dohme et al., 2020). As suggested by Landin and Hebert (1999), we may postulate that the use of self-talk could increase the self-confidence of the participants of the MI + self-talk group inducing greater service efficiency and points won than the participants of the other groups. In addition, Hardy (2006) evoked that motivational self-talk can proactively and reactively regulate motivation and emotion and sustain the effort, which can give an advantage to tennis players during competition. Indeed, Van Raalte et al. (1994) showed that the self-talk (e.g., positive verbalizations) was related to successful game outcome for the servers. Finally, the fact that the MI + self-talk group did not improve more than the MI group, in the percentage of service success, could be explained by a plateau effect due to the level of expertise of the participants limiting the margin of progress. More research is needed to better explore the potential differential effect of MI + self-talk on the percentage of success and tennis technical efficiency.

## Limitation

This study is not without limitations. Firstly, the fact that there was an absence of self-talk only could be considered as a limit to the current study. Indeed, although all participants who beneficiated from MI interventions increased their service performances, with more significant effect for the participants in the MI + self-talk group, it is possible that the use of motivational self-talk alone could be beneficial, even optimal. More research is needed to compare the performance of the participants in all these conditions. In addition, although the video of the participants of the Control and MI groups did not show the use of external observable verbalizations or negative gestures during the post-tests, it is possible that they used internal negative verbalizations that can decrease the probability of increased performance (Van Raalte et al., 2014). In addition, this study was centred on the first service, but due to its natural stress and anxiety, it could be interesting to explore the effect of MI + self-talk on second service performance. Finally, the fact that only skilled players were used in this study can also be seen as a limitation.

## Conclusion

The current study highlights the beneficial effect of using a combination of MI and self-talk to improve the service performance in skilled players and provides additional arguments in favour of mental imagery in tennis. Although the results obtained in the current must be confirmed, it seems that the combination of motivational self-talk and MI, performed before serving, can be beneficial in tennis players. We suggest expert players to test and choose individual motivational self-talk, in training, and to combine it with MI, for later use in matches. More research is needed to understand better and explore the effect of MI and self-talk, in different tennis task performances, especially with participants of varying skill levels.

## Data Availability Statement

The raw data supporting the conclusions of this article will be made available by the authors, without undue reservation.

## Ethics Statement

The studies involving human participants were reviewed and approved by ACTES (EA3596), Université des Antilles. The patients/participants provided their written informed consent to participate in this study.

## Author Contributions

All authors listed have made a substantial, direct, and intellectual contribution to the work and approved it for publication.

## Conflict of Interest

The authors declare that the research was conducted in the absence of any commercial or financial relationships that could be construed as a potential conflict of interest.

## Publisher’s Note

All claims expressed in this article are solely those of the authors and do not necessarily represent those of their affiliated organizations, or those of the publisher, the editors and the reviewers. Any product that may be evaluated in this article, or claim that may be made by its manufacturer, is not guaranteed or endorsed by the publisher.
